# Right coronary artery dissection after aortic valve replacement presenting with refractory ventricular fibrillation

**DOI:** 10.1093/jscr/rjad717

**Published:** 2024-01-16

**Authors:** Abiah Jacob, Natalia Hara, Giridhara Goli, Kulvinder Lall

**Affiliations:** Barts Heart Centre, St. Bartholomew’s Hospital, West Smithfield, EC1A 7BE London, United Kingdom; Barts and the London School of Medicine and Dentistry, Queen Mary University of London, E1 2AD London, United Kingdom; Barts Heart Centre, St. Bartholomew’s Hospital, West Smithfield, EC1A 7BE London, United Kingdom; Barts Heart Centre, St. Bartholomew’s Hospital, West Smithfield, EC1A 7BE London, United Kingdom

**Keywords:** cardiac surgery, aortic valve replacement, coronary artery dissection, infective endocarditis, ventricular arrhythmias

## Abstract

Iatrogenic coronary artery dissection is a rare complication seen in 0.07% of coronary procedures. Presentations of this condition vary, ranging from signs of myocardial ischemia to rarer presentations of ventricular arrhythmias. We present a rare case of a 55-year-old patient with native aortic valve endocarditis who developed right coronary artery dissection (RCAD) in the immediate post-op period presenting with refractory ventricular fibrillation (VF). Emergency coronary angiogram revealed an extensive RCAD extending from the ostium to the mid-vessel as the cause of VF. A consensus between the cardiologists and the cardiac surgeons led to an emergency right coronary artery bypass graft (CABG) that resolved the VF. This case illustrates a rare presentation of iatrogenic RCAD and the successful management of the same. We highlight the importance of prompt detection via angiography in patients suspected of having coronary artery dissection and showcase the successful implementation of emergency CABG in a patient with unstable haemodynamics.

## Introduction

Iatrogenic coronary artery dissection (ICAD) is a rare complication seen in 0.07% of coronary procedures, related to percutaneous coronary intervention (PCI) and antegrade cardioplegia delivery [1, 2]. Presentations of this condition vary, ranging from signs of myocardial ischemia like chest pain, dyspnoea and diaphoresis, to rarer presentations of ventricular arrhythmias. A high index of suspicion is required to diagnose this condition especially in intubated and ventilated patients. We present a rare case of a patient with native aortic valve endocarditis who developed right coronary artery dissection (RCAD) in the immediate post-operative period, presenting with refractory ventricular fibrillation (VF).

## Case report

**Figure 1:**
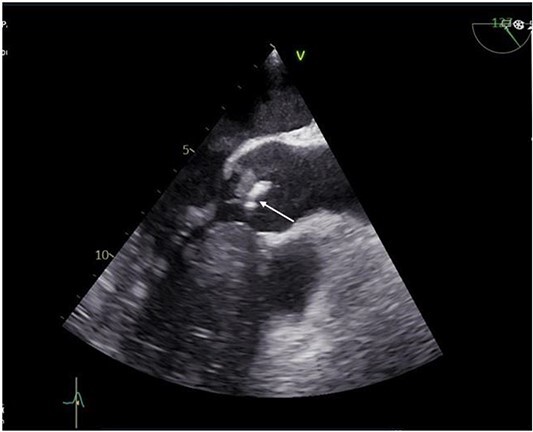
Preoperative transthoracic echocardiogram (TTE) showing vegetation on the aortic valve.

A 55-year-old gentleman presented to the emergency department with a 5-month history of ‘feeling unwell’, associated with weight loss, night sweats, cough, and lower limb weakness. He was treated as an outpatient for 3 weeks with antibiotics but had no respite and was eventually admitted to the hospital due to persistent symptoms. Transoesophageal echocardiography (TOE) showed a 10 mm vegetation prolapsing into the left ventricular outflow tract, attached to a bicuspid, calcified aortic valve, with severe aortic regurgitation (Fig. 1). He had an aortic valve area of 2.0cm [2] and left ventricular ejection fraction of 65%.

Blood cultures showed positive microbial growth with *Streptococcus Mutans*, leading to a diagnosis of native bicuspid aortic valve infective endocarditis for which he received intravenous antibiotics. He had no peripheral stigmata of infective endocarditis and auscultation revealed a diastolic murmur in the aortic region. Computerized tomography (CT) coronary angiogram excluded obstructive coronary artery disease.

**Figure 2:**
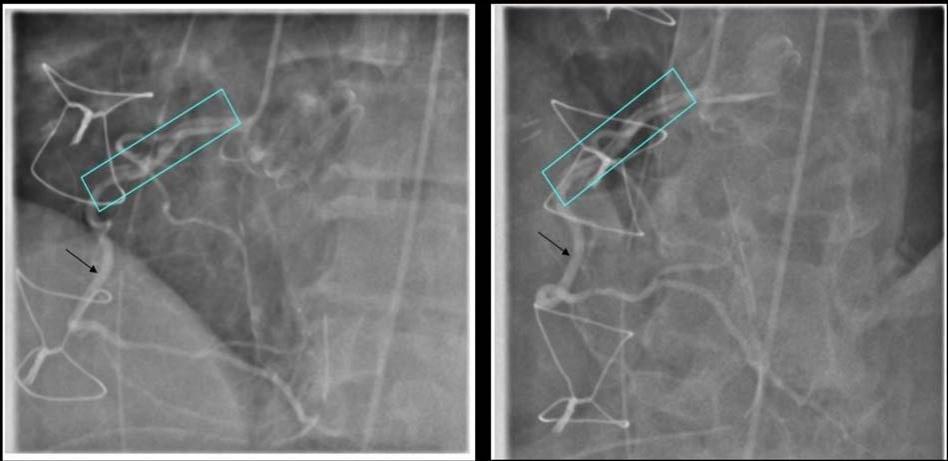
Coronary Angiogram showing RCA dissection extending from ostium to mid-vessel (box), normal coronary artery (arrow).

He underwent a successful urgent mechanical aortic valve replacement (25 mm On-X mechanical valve) on cardiopulmonary bypass (CPB) with cardioplegic arrest. Cardioplegic protection was achieved using antegrade intermittent cold blood. Intra-operatively, a severely calcified functional bicuspid aortic valve with commissural fusion between the right and left cusps was seen. The aortic tissue was noted to be fragile. At the end of the procedure he developed atrial fibrillation which reverted to sinus tachycardia on administration of Amiodarone. His aortic cross clamp time was 91 min and bypass time was 116 min. He was successfully weaned off CPB and safely transferred to the intensive care unit (ITU). On arrival to the ITU, he went into refractory VF requiring 12 attempts of electrical cardioversion, amiodarone infusion and lidocaine, with a blood gas lactate of 5.3. His ECG revealed inferior and lateral ST depression and TOE showed inferior wall hypokinesia. An emergency coronary angiogram revealed an extensive right coronary artery (RCA) dissection extending from the ostium to the mid-vessel as the cause of VF (Fig. 2).

Multidisciplinary team consensus between the cardiologists and the cardiac surgeons led to an emergency RCA bypass graft (CABG). Post-operatively, he was transferred to the ITU in a stable condition. He developed Dressler’s syndrome whilst recovering and was successfully treated for the same. Post-operative echocardiogram (ECHO) showed a well seated aortic valve without valvular vegetations. He was discharged home on long term antibiotics. On follow-up, he is active and well in himself with no cardiac symptoms.

## Discussion

Aortic valve replacement can be performed with either retrograde or antegrade cardioplegia. We preferred using antegrade cardioplegia to ensure right ventricular protection, which is achieved by delivering cardioplegia either indirectly to the aortic root or selectively to the coronary artery ostium. One of the rare complications related to antegrade cardioplegia delivery is coronary artery dissection [1, 2].

Coronary artery dissection can be spontaneous, traumatic, or iatrogenic and is due to the separation of the coronary artery wall layers causing blood to enter the intimal space. Presentation is similar to symptoms and signs of an acute myocardial infarction (MI) such as chest or shoulder pain, syncope, dyspnoea, diaphoresis, and nausea associated with elevation of cardiac enzymes. However, a small proportion (3–14%) of patients present with ventricular arrhythmias like our patient who had refractory VF [3]. Spontaneous coronary artery dissection is the sudden development of a false lumen within the coronary artery wall that may compromise coronary blood flow by externally compressing the true lumen and is a recognized, rare cause of sudden cardiac death, possibly as a result of ventricular arrhythmia triggered by myocardial ischaemia or infarction [4].

ICAD is rare and seen in 0.07% of coronary procedures, occurring twice as likely during PCI versus diagnostic catheterization. The dissection results from mechanical injury to the arterial wall during catheter or wire manipulation, passage or deployment of an interventional device, balloon dilatation and stenting or forceful injection of contrast medium [5–6]. Another iatrogenic cause is during instrumentation with the antegrade cardioplegia catheter during cardiac surgery. In this case, the intima of the vessel is disrupted, causing dissection of tissues that can propagate into the ascending aorta, the arch or around the pericardium, causing cardiac tamponade [7]. Our patient received anterograde cardioplegia intra-operatively through the coronary ostia and we believe that instrumentation during cardioplegia delivery is the cause of his RCAD.

ICAD should be considered in post-operative patients who present with signs of myocardial ischemia and have undergone coronary perfusion via the coronary ostia. Due to the rarity of this condition after cardiac surgery, it entails a high degree of suspicion for prompt diagnosis and treatment especially in intubated and ventilated patients who may present with biochemical, ECG or imaging signs of myocardial ischemia such as raised lactate, regional wall motion abnormalities on echocardiography, refractory ventricular arrythmias, and haemodynamic instability as seen in our patient. Emergency angiography is indicated in these patients followed by either PCI or emergency CABG depending on the lesion. In a haemodynamically unstable patient, rapid revascularization may be needed [8]. Strategies for the treatment of iatrogenic dissection are: conservative therapy, stent implantation, and CABG [9–11]. Due to the unpredictable nature of the dissection flap and the potential for disastrous sequelae, conservative therapy is scarcely considered in patients with iatrogenic dissection. However, in a study by Eshtehardi *et al*., conservative medical management was chosen in selected patients with localized and stable dissections and was linked with a favourable safety profile during follow-up. Nonetheless, in most patients, a strategy to seal the dissection flap with a stent or a CABG was preferred [5].

In our patient, a multidisciplinary decision was made to perform an emergency CABG instead of a PCI, since the dissection extended from the right coronary ostium. This is in contrast to reports that suggest the use of stents instead of high-risk emergency surgery, stating the rationale that although emergency CABG is effective, it is time consuming and risks irreversible myocardial damage [12]. However, our patient had a successful outcome.

This case highlights a rare presentation of ICAD and the successful management of the same. It illustrates the importance of prompt detection via angiography in patients suspected of having ICAD and showcases the successful implementation of emergency CABG in a patient with unstable haemodynamics. As a result of our experience, we recommend using a soft, balloon-tipped catheter to deliver antegrade cardioplegia through the coronary ostia rather than rigid tipped catheters in patients with fragile tissues, such as the elderly and those with endocarditis, in order to prevent traumatic iatrogenic injuries. Furthermore, to mitigate and overcome complications, multidisciplinary team collaboration is imperative and these cases need to be managed at centres of excellence.

## Data Availability

The data underlying this article are available within this article.
